# Cost Effectiveness Analysis Using Disability-Adjusted Life Years for Cataract Surgery

**DOI:** 10.3390/ijerph17166010

**Published:** 2020-08-18

**Authors:** Thinni Nurul Rochmah, Anggun Wulandari, Maznah Dahlui, Ernawaty, Ratna Dwi Wulandari

**Affiliations:** 1 Faculty of Public Health, Universitas Airlangga, Surabaya 60115, Indonesia; maznahd@ummc.edu.my (M.D.); ernawaty@fkm.unair.ac.id (E.); Ratna-d-w@fkm.unair.ac.id (R.D.W.); 2 Faculty of Medicine, Universitas Lambung Mangkurat, South Kalimantan 70123, Indonesia; anggun.wulandari-2017@fkm.unair.ac.id; 3 Faculty of Medicine, University of Malaya, Kuala Lumpur 50603, Malaysia

**Keywords:** cataract surgery, cost effectiveness analysis, disability adjusted life years

## Abstract

Cataracts are the second most prioritized eye disease in the world. Cataracts are an expensive treatment because surgery is the only method that can treat the disease. This study aims to analyze the cost effectiveness of each operating procedure. Specifically, phacoemulsification and Small Incision Cataract Surgery (SICS) with Disability-Adjusted Life Years (DALYs) as the effectiveness indicator is used. This study is an observational analytic study with a prospective framework. The sample size is 130 patients who have undergone phacoemulsification and 25 patients who have undergone SICS. The DALY for phacoemulsification at Day-7 (D-7) is 0.3204, and at Day-21 (D-21), it is 0.3204, while the DALY for SICS at D-7 is 0.3060, and at D-21, it is 0.3158. The incremental cost effectiveness ratio (ICER) for cataract surgery at D-7 is USD $1872.49, and at D-21, it is USD $5861.71, whereas the Indonesian Gross Domestic Product (GDP) is USD $4174.90. In conclusion, the phacoemulsification technique is more cost effective than the SICS technique. The ICER value is very cost effective at D-7 post-surgery compared to at D-21 post-surgery because the ICER is less than 1 GDP per capita per DALY.

## 1. Introduction

A cataract is a state where an eye lens that is usually clear and transparent becomes murky [1]. If the lens loses its transparent and clear nature, then vision will turn hazy, which eventually leads to blindness. A cataract is indicated by an eye lens that gradually becomes blurry, and the condition can lead to total blindness. Cataracts are mostly linked to degeneration processes related to age. Nowadays, cataracts are the most dominant eye disease and the main cause of blindness worldwide [2].

The prevalence of cataracts in East Java Province based on Basic Health Research or Riskesdas (Riset kesehatan dasar) n 2013 was 1.6%. Even though cataract prevalence in East Java is under the national standard (1.8%), the number of people with blindness in East Java in 2013 is estimated to be 141,132 people. This number represents the second-highest proportion of the population with blindness by Province in Indonesia, after Central Java [3]. Every year, as many as 38,000 residents of East Java are at risk in contracting cataracts [4].

In line with the high cataract prevalence in East Java, the cataract data obtained from the eye center Hospital “Rumah Sakit Mata Undaan” (RSMU) in Surabaya show a highly significant increase in cases in the last 3 years. RSMU is one of the eye Hospital centers in Surabaya. In RSMU, cataracts were always included in the top ten most prevalent diseases from 2015 to 2017, with an average of 7856 cases (20.50%) in a time period.

Cataracts require a huge cost for treatment. According to Health Social Insurance Administration Organization or BPJS Kesehatan (Badan Penyelenggara Jaminan Sosial Kesehatan) data, the cost of treating cataract disease in a year has reached USD $2.6 trillion. This number is larger than the cost of treating catastrophic diseases, which cost only USD $2.3 trillion a year [5]. Besides affecting the economic burden of health care, various research projects also show that visual impairment has a negative impact on mobility, participation in social activities, ability to work, the ways people spend their free time, and the ability to do daily activities, causing people to become dependent. It is also linked to depression [6]. People who have cataracts usually lose potential days of work. The estimated loss in the form of losing potential days of work in a certain number of years due to cataracts in macroeconomics is called disability-adjusted life years (DALYs) [7]. Cost-per-DALY studies focus mostly on countries in Africa and Asia. DALYs were developed to measure the global burden of disease and were popularized in the global health community [8].

Cataracts can only be treated by surgery [9]. As yet, there are no medicines, foods, or sport activities that may prevent or cure a person from cataracts. There are various methods of correcting cataracts surgically, e.g., Intra Capsular Cataract Extraction (ICCE), Extra Capsular Cataract Extraction (ECCE), phacoemulsification, and Small Incision Cataract Surgery (SICS) [1]. Three methods of surgery were used at the RSMU for cataract surgery in January–November 2018. During that period, the most widely used surgical action was phacoemulsification, which represented 88.28% of cases, SICS represented 10.08%, and ECCE represented 1.53%.

The variation in types of surgery will result in differences in costs. Thus, to determine the cost effectiveness of inter-action use, a type of analysis called Cost Effectiveness Analysis (CEA) is needed [10]. CEA is a way to choose and assess the best course of treatment or action if there are several choices with the same goal [11].

With CEA, the cost of an alternative viewed from a certain perspective is compared to the health improvement gained from the alternative, where improvement in health is measured in DALYs that can be eliminated because of an action. The results are stated as cost per DALY. This method is an economic evaluation method that can be used for decision making to choose the best alternative from several alternatives [12]. In this study, the alternative actions to be compared are phacoemulsification and SICS.

Phacoemulsification is a type of minimal stitching cataract surgery that uses ultrasonic vibrations to destroy the lens’ nucleus. Phacoemulsification is far more dependent on technology than other surgery techniques. Phacoemulsification is a cataract extraction technique using small incisions of about 1.5 to 3 mm with implantation of a foldable intraocular lens, so that wound closure can be done without stitches [13]. Boughton [12] stated that in relation to treatment, costs incurred using phacoemulsification techniques; policy analysts have questioned the feasibility of utilizing phacoemulsification techniques in countries with lower–middle incomes. Phacoemulsification has now become a mainstream treatment for cataracts. Phacoemulsification is preferred in developed countries with large health care budgets [14].

Small Incision Cataract Surgery (SICS) is another surgery method. This technique is not machine-dependent, does not require expensive investment equipment, and is relatively easy to teach to novice operators. These are some of the reasons why the use of the SICS technique is being considered a safe and effective technique for cataract surgery, especially in developing countries [15]. Moreover, SICS has several advantages, including better wound stability and refractive stability due to small 5–6 mm wound incisions, patient comfort due to faster visual healing, minimal chance of collapse of the intra-operative front part of the eye, and minimal other intra-operative complications and minimal post-surgery visits [16]. The difference between the two methods gives different DALY results, where a higher DALY score represents a worse patient health status.

The economic assessment (e.g., cost-effectiveness analysis using Disability-Adjusted Life Years, DALYs) of interventions focusing on cataract surgery is helpful for evaluating the effectiveness of interventions. With CEA, the cost of an alternative viewed from a certain point of view is compared with the health improvement gained from that alternative. Health effects in terms of global health are typically measured as inverted adjusted life-years (DALYs). The results are expressed as cost per DALY, which is referred to as the Incremental Cost Effectiveness Ratio compared to the threshold value, as a benchmark. Because an incremental cost effectiveness ratio (ICER) can be thought of as the “price” at which an intervention produces health gains, that price must be compared with a benchmark to determine whether the intervention is “cost-effective” [17].

ICERs exceeding the threshold are considered unfavorable. Meanwhile, the threshold represents what society is willing to pay for health gains and, correspondingly, what goods and services it would be willing to forego for these gains, its assumed importance. If total health care spending is fixed, implying that other health care interventions must be eliminated to pay for the adoption of a new intervention, the new intervention’s ICER should be at least as favorable as the ICER for interventions that are cut [17].

Referring to the World Health Organization’s Choosing Interventions that are Cost–Effective (WHO-CHOICE) project, an intervention is very cost-effective if its incremental cost-effectiveness ratio (ICER) is less or equal 1 Gross Domestic Product (GDP) per capita per DALY, cost-effective if it ranges from 1 to 3, and not cost-effective if it is greater than three GDP per capita per DALY [18].

To the best of our knowledge, there are no published studies comparing the effectiveness of phacoemulsification and SICS treatments for cataract surgery in Surabaya, Indonesia. The aim of this study is to evaluate, from a cost-effectiveness perspective, two different surgery procedures: phacoemulsification and SICS.

## 2. Materials and Methods

This study was an observational analytic study with a quantitative approach. It was conducted at the “Rumah Sakit Mata Undaan” (RSMU) hospital in Surabaya. RSMU was chosen as the location of the study because this hospital serves most of the general cataract surgery patients who undergo phacoemulsification and SICS in the city of Surabaya. Research activities were carried out in January–March 2019. The participants in this study were patients with out-of-pocket payments, who underwent cataract surgery at the “Rumah Sakit Mata Undaan” hospital in Surabaya. They were assessed on Day 7 (D-7) post-surgery and Day 21 (D-21) post-surgery. D-7 and D-21 post-surgery were chosen because the average recovery time of phacoemulsification patients is at D-7 post-surgery, whereas the average recovery of SICS patients is at D-21 post-surgery. Thus, a comparison of D-7 and D-21 was used to see the overall difference between the two surgical techniques.

The inclusion criteria in this research were patients with mature cataracts, patients without complications of glaucoma, retinal detachment, and traumatic cataracts. Calculation of the sample size in this study was done using the Lemeshow formula. The Lemeshow formula was used based on a database at the “Rumah Sakit Mata Undaan” hospital in Surabaya last year, and the researchers obtained data from 130 patients who underwent the phacoemulsification technique and 25 patients who underwent SICS. This was a prospective study, which was done by following patients before surgery until 21 days post-surgery. The sampling process was accidental sampling.

The calculated costs consisted of direct costs and indirect costs. Direct costs were the costs needed to finance cataract treatment at the hospital. Indirect costs consisted of transportation costs, costs due to lost productivity, and caregiver costs. Direct cost data were determined using secondary data from the hospital, while indirect cost data were collected through a questionnaire involving interviews.

All subjects gave their informed consent for inclusion before they participated in the study. The study was conducted in accordance with the Health Research Ethics Committee, Faculty of Nursing, Universitas Airlangga with approval (ref number: 1265-KEPK). This study used Microsoft Excel to calculate the financial and DALY data, while an independent T-test was used to compare the incomes of the phacoemulsification group and SICS group.

## 3. Results

### 3.1. Cataract Surgery Cost

Based on the results of the calculation of pre-surgery costs, surgery costs, and post-cataract surgery costs, the total direct surgery cost was calculated, including the minimum and maximum direct costs. The direct cost per capita of cataract surgery using surgery phacoemulsification was USD $659.56 at Day 7 (D-7) post-surgery and USD $677.09 at Day 21 (D-21) post-surgery. Meanwhile, the direct cost per capita of cataract surgery using SICS was USD $640.61 at Day (D-7) post-surgery and USD $666.57 at Day-21 (D-21) post-surgery (Table 1). This can be interpreted as showing that the highest total direct cost of cataract surgery on D-7 and D-21 was cataract surgery using the phacoemulsification technique.

Based on the calculation of transportation costs, the cost of lost productivity, and caregiver costs, the total indirect surgery costs were calculated, including the minimum and maximum costs. In addition, to compare the lost productivity cost at D-7 and D-21 post-surgery, an independent T-test was done, and the results showed that no significant difference (*p* > 0.05) between the incomes of SICS and phacoemulsification patients. Productivity loss was used because it was relatively similar between phacoemulsification patients and SICS patients; therefore, productivity loss was only influenced by the length of illness. The indirect cost of cataract surgery per capita in patients using the phacoemulsification technique was USD $82.82 at D-7 post-surgery and USD $95.84 at D-21 post surgery. On the other hand, the indirect cost of surgery per capita for patients using the SICS technique was USD $68.94 at D-7 post-surgery and USD $79.40 at D-21 post surgery (Table 2). However, the trend for indirect costs was the same as for direct costs: the costs of phacoemulsification at D-7 post-surgery and at D-21 post-surgery were higher than for SICS.

Based on the calculation of direct and indirect costs, the average total overall cost was obtained. The average direct and indirect costs of cataract surgery per capita in patients who underwent the phacoemulsification technique was USD $742.38 at D-7 post-surgery and USD $772.93 at D-21 post-surgery. Furthermore, the average direct and indirect costs of cataract surgery per capita in patients who underwent the SICS technique was USD $709.55 at D-7 post-surgery and USD $745.97 at D-21 post-surgery (Table 3). As a result, the total cost incurred for cataract surgery by patients was greater when the phacoemulsification technique was used compared to when the SICS technique was used.

### 3.2. Disability-Adjusted Life Years (DALYs)

Disability-Adjusted Life Years (DALYs) is a measure of the disease burden expressed in the number of years lost due to illness by adding up years of life lost (YLL) and years lived with disability (YLD). Years of life lost (YLL) is an estimation of the number of years of life lost due to early death by multiplying the number of cases and life expectancy at death. However, cataract cases do not cause death so the YLL is considered to be zero. The years lived with disability (YLD) is an estimation of the years of life lived with a disability by multiplying the disability weight and the duration from illness to recovery. The DALY value on D-7 and D-21 for phacoemulsification was constant because the patients had recovered at D-7 post-surgery so the DALY value did not change, while the SICS patients recovered at D-21, so the DALY value increased (Table 4). Based on DALY calculations, the results were as follows:

### 3.3. Incremental Cost Effectiveness Ratio (ICER)

The ICER is a benchmark that is used to compare the DALY value with the threshold in a country to get an illustration of the effectiveness of cataract surgery using the phacoemulsification technique and the SICS technique. ICER is obtained by dividing the difference between the average cost of phacoemulsification and the average cost of SICS with the difference between the DALYs of phacoemulsification and SICS at D-7 and D-21. Based on the calculation, the ICER at D-7 was USD 1872.49, and at D-21, it was USD 5861.71. The GDP of Indonesia is 4174.90, and WHO-CHOICE [18] recommends that the ICER value is less than or equal to 1 GDP per capita per DALY. A treatment is cost-effective if the ICER value ranges from 1 to 3 and not cost-effective if it is greater than three GDP per capita per DALY. Thus, the ICER value was compared with the threshold value of less than 1 GDP at D-7 (USD 1872.49), which means very cost effective. On the contrary, the ICER value at D-21 ranges from 1 to 3 (USD 5861.71) which shows that the treatment is cost effective enough (Table 5).

## 4. Discussion

To the best of our knowledge, this is the first study to conduct a cost effectiveness analysis using DALYs for cataract surgery. This quantitative study may be useful for the evaluation of specific health policies focused on the selection of cataract surgery techniques that are more cost effective.

Our findings show that the direct cost of post-surgery control in patients using phacoemulsification techniques is greater than the direct cost of post-surgery control in patients who undergo the SICS technique. This is because, at the time of post-surgery control, patients who undergo the phacoemulsification technique incur greater costs compared with patients who undergo SICS.

Furthermore, based on interviews with ophthalmologists, patients can work again after 7 days and 21 days post-surgery, as the recovery from phacoemulsification is faster than from SICS. Therefore, patients who undergo cataract surgery who undergo phacoemulsification are able to work again at D-7 post-surgery, but patients who undergo SICS are only allowed work at D-21 post-surgery. This is because phacoemulsification does not require stitches, while SICS requires stitches. In addition, the process of surgery with phacoemulsification is faster, the risk of infection and bleeding is smaller, the incision is small, and the treatment does not cause pain. In contrast, for SICS, the operation process is longer, the risk of infection and bleeding is greater, the incision is wider, and more aches and pains occur.

In the post-surgery period, patients are prohibited from lifting weights >10 kg, the eyes must not be exposed to water, the patient may not bend down for too long, and the patient must not be exposed to smoke and dust. According to ophthalmologists interviewed, this advice is too strict for patients with phacoemulsification, because the risk of complications in phacoemulsification patients is smaller. In contrast, in SICS patients, this advice is very important given the risk of damage to the eye stitches. In this study, all standard operating procedures were followed, and the patients were compliant with existing medical procedures; therefore, the researchers were able to more extensively look at the cost aspect.

Based on the results of the calculation of pre-surgery costs, surgery costs, and post-surgery costs, the total direct surgery costs were calculated. The direct cost of surgery was lower when the SICS technique was used compared with the phacoemulsification technique. Indirect costs included costs due to lost productivity, transportation costs to travel to the health care setting, caregiver costs, and cost of assistive devices used during the treatment. The lost productivity cost included money lost due to the loss of productive time of patients and their companions during treatment. The cost of lost productivity was measured by calculating the lost working time (in hours or days) multiplied by the revenue that should have been obtained (in hours or days) or the number of products that were not successfully produced or sold multiplied by the unit price of the product. In addition, lost productivity in this study was compared at D-7 post-surgery and D-21 post-surgery by an independent T-test, and the results showed that there was no significant difference (*p* > 0.05) between the income of SICS and phacoemulsification patients.

Transportation costs included the transportation costs incurred by patients and their companions to go to the place of health care during treatment. Transportation costs were calculated by knowing the means of transportation used by the patient and the distance of each patient’s residence to the “Rumah Sakit Mata Undaan” hospital. For patients who used a motorcycle, it was estimated that respondents would use 1 L of fuel for 51 km. We multiplied the distance by the price of 1 L of fuel. For cars, it was estimated that 1 L of fuel would be used for 8.9 km [14]. The cost of public transportation was acquired by asking the patient. Transportation costs for online motorcycles and online taxis were acquired by using the provider application.

Caregiver costs are costs incurred by the companions of cataract patients as remuneration for providing company or escorting them to the place of health care during treatment. Based on the results of the calculation of lost productivity costs, transportation costs, caregiver costs, and the cost of assistive devices, the total indirect surgery cost was calculated. The average indirect cost incurred by phacoemulsification patients was greater than that of SICS patients. This was due to the lost productivity costs and transportation costs of phacoemulsification patients, which were greater than those of SICS patients.

DALYs are used in scientific techniques to estimate the comparative magnitude of health loss due to diseases and risk factors by age at specific points in time. DALYs are used to estimate health loss and can be measured through premature deaths (YLL) and/or non-fetal disability (YLD) [19]. The number of Disability-Adjusted Life Years (DALYs) in patients treated with the phacoemulsification technique was greater than in patients treated with SICS, i.e., the magnitude of the number of years lost due to disability due to cataracts in patients treated with SICS is smaller than that of patients treated with phacoemulsification. However, patients treated with the phacoemulsification technique had recovered more at D-7 post-surgery than those treated with the SICS technique. Currently, the most common surgical method used to treat cataracts is phacoemulsification, because it can be used at any stage of cataract development [20]. Therefore, SICS patients need more days to heal and only recover at D-21 post-surgery, due to the larger incision made than in the phacoemulsification technique.

In addition, the recovery of patients that had occurred on D-21 still showed good results because phacoemulsification patients had recovered on D-7, while SICS patients needed additional time to recover at D-21. The ICER of recovering at D-7 is more cost effective than that of recovering on D-21, so the phacoemulsification technique is more cost effective than SICS, at both D-7 and D-21. This is because, based on WHO-CHOICE recommendations (2014), the ICER of interventions can be measured by comparing threshold values measured as the GDP per capita versus the DALY value. If the GDP is less or equal to 1, then the treatment is very cost effective, if it is from 1 to 3, then the treatment is quite effective, and if the value is greater than three GDP per capita per DALY, the treatment is not effective.

This is in line with research conducted by Shandya [21], which recommended the use of phacoemulsification to treat cataracts. Moreover, our results are in accordance with the results of another study conducted by Anggun [22], which stated that cataract surgery with the phacoemulsification technique is more cost-effective than using the SICS technique.

Based on guidance, the threshold value in Indonesia is USD $4174.90, which means that the ICER value of cataract surgery at D-7 (USD $1872.49) represents greater cost effectiveness than at D-21 (USD $5861.71). However, phacoemulsification and SICS can both be cost effective, depending on the threshold value.

## 5. Conclusions

The phacoemulsification technique was shown to be a more effective technique than the SICS technique when DALYs were used to measure the effectiveness. At D-7 post-surgery, patients who underwent the phacoemulsification technique showed increased recovery compared with those who underwent the SICS technique. The cure rate for SICS was equals to the cure rate for phacoemulsification techniques at D-21 post-surgery. In conclusion, the ICER at D-7 was less than 1 GDP, USD $1872.49, showing greater cost effectiveness compared with the value at D-21 of USD 5861.7, which is more than 1 GDP, representing that it is cost effective enough.

## 6. Patent

This research included non-patented works because further research can find other differences in results.

## Figures and Tables

**Table 1:** Direct Costs of Cataract Surgery Per Capita at Day 7 (D-7) and Day 21 (D-21).

Surgery Technique	Direct Costs (USD)									
	Pre-Surgery Cost	Surgery Cost	Post-Cataract Surgery Cost		Total Direct Cost (USD)		Minimum Direct Cost (USD)		Maximum Direct Cost (USD)	
			D-7	D-21	D-7	D-21	D-7	D-21	D-7	D-21
**Small Incision Cataract Surgery (SICS)**	27.63	562.18	50.80	76.76	640.61	666.57	319.93	319.93	818.93	859.93
**Phacoemulsification**	25.18	595.01	39.37	56.90	659.56	677.09	436.14	437.64	1280.43	1302.64

D-7, Day 7; D-21, Day-21.

**Table 2:** Indirect Costs of Cataract Surgery Per Capita at Day 7 (D-7) and Day 21 (D-21).

Surgery Technique	Indirect Costs (USD)											
	Lost Productivity Costs		Transportation Costs		Caregiver Costs		Total Indirect Cost (USD)		Minimum Indirect Cost (USD)		Maximum Indirect Cost (USD)	
	D-7	D-21	D-7	D-21	D-7	D-21	D-7	D-21	D-7	D-21	D-7	D-21
**SICS**	38.65	43.87	26.53	31.48	3.76	4.04	68.94	79.40	1.53	1.91	247.62	309.52
**Phacoemulsification**	49.18	55.43	31.45	38.15	2.20	2.26	82.82	95.84	0.47	2.36	807.00	1161.76

SICS—Small Incision Cataract Surgery.

**Table 3:** Total Cost of Cataract Surgery Per Capita at Day 7 (D-7) and Day 21 (D-21).

Surgery Technique	Total Costs (USD)											
	Direct Costs		Indirect Costs		Total (USD)		Average (USD)		Minimum Total Cost (USD)		Maximum Total Cost (USD)	
	D-7	D-21	D-7	D-21	D-7	D-21	D-7	D-21	D-7	D-21	D-7	D-21
**SICS**	16,015.29	16,664.29	1723.55	1984.90	17,738.83	18,649.18	709.55	745.97	322.54	405.76	997.98	1068.24
**Phacoemulsification**	85,742.86	88,022.25	10,767.16	12,458.82	95,510.01	100,481.07	742.38	772.93	441.75	459.45	1287.62	1958.83

SICS—Small Incision Cataract Surgery.

**Table 4:** Number of Disability-Adjusted Life Years (DALYs) Per Capita.

Surgery Technique	DALYs per Capita D-7	DALYs per Capita D-21
**SICS**	0.3060	0.3158
**Phacoemulsification**	0.3204	0.3204

D-7, Day 7; D-21, Day-21; SICS—Small Incision Cataract Surgery.

**Table 5:** Distribution Frequency of the *Incremental* *Cost Effectiveness Ratio* (ICER).

Variable	Surgery Technique		
	Phacoemulsification	SICS	Incremental (Phacoemulsification-SICS)
**Average Total Cost (USD)**	772.93	745.97	26.96
**DALY D-7**	0.3204	0.3060	0.0144
**DALY D-21**	0.3204	0.3158	0.0046
**ICER D-7** =			1872.49
**ICER D-21** =			5861.71

D-7, Day 7; D-21, Day-21; SICS- Small Incision Cataract Surgery; DALYs - Disability-Adjusted Life Years.
